# Medical Miscellanies

**Published:** 1816-03

**Authors:** 


### PART IV.

### MEDICAL MISCELLANIES.

### *Fatal Lesion of the Heart, with the Appearances on Dissection.**

A French captain from Versailles, aged 45, of middle stature and regular make; his head large; neck short; chest broad and

* Hufeland's *Journal der Practischen Heilkunde.* The translation is not literal; but all the important facts are stated with fidelity.

well arched, with the ensiform cartilage bent inwards; and arms of a length scarcely proportioned to the trunk; came, in January 1813, as prisoner of war into the hospital, on account of a frost-bitten foot, which required amputation at the tarsus. In other respects the patient was healthy. During twenty years' service, he had never suffered a serious illness.

But his unhappy situation, as prisoner of war, affected him deeply. He passed whole nights without sleep, and the days were spent, in a state of mournful reverie, upon the bed. The wound went on favourably for a time, and was nearly cicatrized; when, in the month of April, he was attacked by a short dry cough and slight oppression of the breast: yet his appetite and countenance continued good, and all the vital functions were regularly performed. The slight paroxysms of cough and dyspnœa were rarely of long duration. With these symptoms there occurred a complete cessation in the healing of the wound, which till then had sensibly advanced. Indeed, the sore again gradually broke open in its whole circumference: the suppuration also ceased; the wound often bled, and was sometimes exceedingly painful. In this state, the patient went on until the end of January 1814, when the writer of this narrative undertook the treatment of the case. The patient now complained of an aggravation of the asthmatic paroxysms; which came on by night, and from severe cold, more frequently than before: he was also obliged to sit upright in bed longer and more frequently, in order to relieve his respiration during the attacks of thoracic oppression.

The secretions and excretions seemed, on the whole, to go on regularly; but the urine was scanty; often voided by drops, and of a very pale colour; sometimes deep red and separating: the fæces costive. The patient, notwithstanding his good appetite, constantly complained of great exhaustion; yet, as observed by himself, he rather gained flesh than lost it. His melancholy was daily aggravated. His fixed dejected look, his livid countenance, his swollen purple lips, and dilated nostrils, bespoke, at the first glance, the severity of his sufferings. A zone of yellowish colour sometimes encompassed his mouth: it came and disappeared with equal rapidity. Towards evening, a dry cough invariably recurred: during the day also the patient was harassed by it; and, later at night, the dyspnœa and oppression of the breast were increased.

An observation was made by the patient himself, that, for some time, his heart had beaten stronger than common; and the vehement pulsation of the jugular vessels* was still more conspicuous. The pulse flagged or intermitted every fifteenth or twentieth stroke; and its variation in strength and velocity was particularly remarkable; alternately low and weak, or full and strong; at one time rapid, at another slow. The wound, which was of a deep red al-

* We suppose that the writer means here the *carotid arteries*. This point should have been more distinctly stated; as, in some species of cardiac lesion, a pulsatory action of the *internal jugular veins* is a very usual attendant.

most black colour, and quite smooth, frequently became very painful, and bled profusely; and, on closer inspection, minute globules of pus, like drops of perspiration oozing from the skin, were seen spread upon it.

As every thing, externally applied to bring back the wound to cicatrization, had hitherto been unavailing, little farther amendment could be expected from other external remedies, especially against the daily aggravated constitutional affection. Wine and cinchona decoction were, however, internally exhibited; and narcotics, particularly opium and aconite, were topically applied, but with little effect; nor were all the attempts at consolation and encouragement more successful. An obtuse pain, coming on in the epigastric region, increased the dejection and distress of the patient. His appetite gradually forsook him. Sometimes there appeared on the legs and breast, small, round, blue spots, like petechiæ, seated somewhat deeply under the skin. They, on one occasion, continued long, gradually disappeared, and then rose again.

April, 1814, was an æra of great political events. The old French soldier heard of them with sensible emotion. He became every day more gloomy and retired; and his appearance and situation grew rapidly and conspicuously worse. In the night of April the 20th, a violent paroxysm of oppression of the breast came on. The patient respired with such difficulty, and felt such agonizing pain about the breast that he thought himself dying. Yet it again went off; and, with the exception of increased debility, his general state remained unaltered. But black spots shewed themselves here and there upon the wound, which must have bled profusely during the night, as all the dressings were stiffened with thick black blood, Night of the 21st. Frequent dry cough; little sleep; wound painful; copious discharge of urine. A blister plaster applied to the breast; and camphor and belladonna internally administered, were productive of relief. The night of the 22d passed quietly without any new incident. On dressing the wound, the dead parts seemed to have been thrown off; and it had a somewhat better appearance. The night of the 23d. Restless; slight paroxysms of thoracic oppression; frequent dry cough and utter sleeplessness. In the morning, the wound had a cadaverous smell, and was of a dark red liver colour: the skin, for some distance around it, was blue. The patient's countenance was more discoloured than ever, much swollen, and the features disfigured: the irregularity of the pulse more decided, unequal in strength and rapidity. On the evening of the 25th, a severe asthmatic paroxysm with violent pressure in the left hypochondrium. This by degrees remitted, and allowed the exhausted patient to slumber for a while. Towards midnight, he was awakened by a profound sense of anguish, and the breast was so constricted that he could scarcely articulate. The paroxysm terminated in death after midnight.

The face, after death, had a dark blue swollen appearance; the eyes were wide open and prominent. In short, all the characters of strangulation were exhibited.

Considering the long residence of this man in the hospital, and his protracted sufferings, the abundance of fat accumulated in the cellular membrane and cavities of the thorax and abdomen, was very remarkable.—The vessels of the *brain* were turgid with blood. —On opening the *thorax*, the dark-blue pericardium presented. Upon nearer examination, however, it might be seen that this appearance was deceptive; the membrane being in reality white. From an incision into the pericardium, which was thickish and readily lacerable, instead of the ordinary serum, almost clear blood gushed out: it was somewhat watery but dark-coloured as venous blood; in quantity, from six to eight ounces. The heart, as it were, swam in it: whence it had issued could not be discovered. The coronary vessels were gorged with blood. The muscular substance of the organ resembled healthy liver rather than heart, in colour and consistence. The adherent pericardium was highly inflamed; and, on incising the heart, a dark-red streak was seen externally on the cut surface, almost as distinct in colour from the substance of the heart itself, as the cortical from the medullary substance of the brain. From the heart's apex to half the extent of its outer surface, a false membrane was deposited, which, like a net, enveloped the organ. Each fibre went off to the internal face of the pericardium, which was otherwise every where loose; and other fibres floated with their loose extremities, like flocculi, in the blood which the pericardium contained. This net-like membrane, in many places, was readily detached with the nail or knife-back; in others, again, it was scarcely, or not at all, separable. The heart itself was considerably enlarged. In the right ventricle was found a pale-red, fleshy, polypus-like substance, nearly occupying half the cavity, and adhering firmly above and below. At its broad end, it was fixed by whitish thick, thread-like fibres near the valves of the ventricle: its pointed end was turned towards the heart's apex. The mass of coagulated blood, which surrounded it, could readily be separated by washing, without in the least destroying this organized substance. It had, throughout, a regular consistence; was tolerably firm and dense, and might be torn in layers from one end to the other. The left ventricle was completely empty; the cavity itself, of the ordinary diameter. But the right ventricle was evidently more capacious than in the natural state. The parietes of the heart were thicker than ordinary; yet they seemed to tear with unusual facility. The auricles were natural; as were also the lungs. The liver enlarged; the gall-bladder full of thick dark-coloured bile; the omentum loaded with fat; the other abdominal viscera healthy. The kidnies were natural; the urinary bladder small, and thickened in its coats.

The comments made upon this interesting case, by the German writer, are not sufficiently important to merit transcription. To us it appears to have been a case of active dilatation of the pulmonary ventricle, complicated with chronic pericarditis; which terminated by effusion of bloody serum into the pericardiac membrane. We recollect once to have met with a similar phænomenon apparently resulting from a like morbid condition of the membrane investing the heart's surface. In the greater number of diversities of cardiac læsion, considerable emaciation of the body is, according to our experience, not commonly met with.

FRACTURE OF THE SKULL WITH DEPRESSION.— On the 27th December, 1815, a boy, eight years of age, was struck and jammed by a very large door, which flew to, with great velocity, impelled by a high wind. An iron staple in the door was the part which struck the right parietal bone, and drove in a piece as large as a dollar, but without incising the teguments. The boy was stunned, but soon recovered from the shock. Medical assistance was immediately procured. The limits of the fracture were clearly defined by the edges of the sound bone all around, while nothing could be felt between, but a soft, hollow, pulsating space, the fractured piece of bone not being tangible. The eyes exhibited nothing unnatural; the senses were clear; the pulse was little altered; in short, there was no symptom of any serious injury to the encephalon, except *sickness at the stomach.* Here, as there was evident and very considerable depression of skull, it became a question whether the elevation of it should not be effected at once, before any bad symptoms arose. The reporter advised *delay*, with copious evacuations, and rigid abstinence. His advice was followed, though the responsibility was considerable. Not a single unpleasant circumstance ensued. A puffy tumor first arose on the fractured space, shewing extravasation under the integuments. This gradually subsided; and in ten days, the fractured bone could be obscurely felt rising towards its original level, and in three weeks the boy returned to school.—The above contains a simple statement of facts, and the writer would feel obliged by the opinion of practical surgeons, whether the circumstances of the case justified his recommending delay. He, himself, on mature reflection, is almost convinced, that " delay was dangerous, " and thinks, he would hardly again give the same counsel, on a similar occasion, notwithstanding the fortunate termination of the case related. — MEDICO-CHIRURGUS.

DEATH FROM A BLOW ON THE STOMACH. — On the 23d of January, 1816, a strong man, 38 years of age, was struck on the abdomen by a large piece of wood from the head of a ship in Portsmouth Dock-yard. He was stunned, and taken to the surgery of the yard, where he was bled, and sent home in a chair. This happened at three o'clock. At six, Mr. Johnson, (one of the Editors) was sent for: Death was then clearly depicted in the man's countenance. The respiration was laborious; the face pale and bluish; the pulse small, quick, and weak; the eyes glassy, the extremities cold. Only a slight scratch was observable about half way between the umbilicus and scrobiculus cordis. The integuments did not appear at all contused. Some bloody urine had been passed; but no fæces. Mr Johnson pronounced the case fatal, and he died in three hours, or six from the accident. — He was opened next day by Mr. Johnson, in presence of Mr. Cowan, one of the surgeons of the Dock-yard. No discoloration of the abdominal parietes was present. On opening the abdomen, some aqueo-sanguineous fluid gushed out, with a portion of air. The transverse arch of the colon was completely sphacelated, black, and almost gelatinous. The cardiac extremity of the stomach was exactly in the same state, and so mortified that it would scarcely bear handling. The bladder was not ruptured, nor any of the intestines. The suddenness of the mortification here is somewhat remarkable. This case shews what injury may be done internally, when little or no external appearance of lesion presents. The conjecture was pretty general among the medical men, *before dissection*, that rupture of the stomach or some other viscus had thus terminated so suddenly the life of the patient. Post mortem inspection corrected this opinion.—J. Johnson.

Vaccination.—The greater uncertainty in the introduction of vaccine than variolous lymph, into the human frame, is well known; and perhaps has a considerable influence in retarding the general adoption of the former. Medical practitioners do not like to be often foiled in producing the vaccine pustule; and parents are not seldom impatient of the delay, during repeated failures, especially when any danger is dreaded from surrounding small-pox. Every hint that may tend to render these failures less frequent will therefore, I should presume, be acceptable. In pursuing the following plan, I have rarely had occasion to introduce vaccine lymph a second time, where it was at all fresh. Let the lymph be taken from a pustule on points of common goose or crow quill, cut as nearly as possible into the shape of a fine spear pointed lancet, at one or both ends. Having made a triangular incision between the cuticle and cutis, slightly grazing the latter, the lancet is to be withdrawn and the point of the vaccinator introduced in its stead, which will prevent any oozing of blood. When held in pretty firmly, for a minute, the superfluous part of the vaccinator is to be snipped off with a pair of scissars, and a small piece of adhesive plaster laid over the wound containing the point of the vaccinator. The next day, the plaster is to be removed, and the point will come with it. In this manner vaccination will succeed in nineteen cases out of twenty, if done in both arms, or in two places in the same arm. — J. Johnson.

*Curious Instance of Metastasis.*—Dr. Parry, in his admirable work on Pathology, just published, has thrown much light on the nature of metastasis, the conversion of diseases, and the salutary effects of even very trifling discharges following determinations of blood to particular parts of the body. The following curious instance has been lately observed by one of the Editors of this Journal. A gentleman is subject to a slight heat, redness, itching, and finally perspiration on the left side of the anus, extending forward to the groin. Whenever, by accident, or any application, this determination to the part disappears, he is seized with a pain in the region of the heart, which continues till the heat, itching, and perspiration near the anus return, when the thoracic pain instantly disappears. Experience has now made him extremely cautious not to check this effort of Nature, and he is very apprehensive, whenever it appears to vanish.

*Apoplexy consequent on Aneurism of both Auricles of the Heart.*—A woman, aged 39, commonly healthy, had experienced, for about a year, from a moral cause, considerable palpitations of the heart, and habitual dyspnœa, much aggravated by walking up an ascent. The pulse was regular, feeble, readily sinking under pressure. The movements of the heart were forcible not extensive. The chest sounded clearly on percussion ; feet œdematous ; all the functions regular. After some days, without any precursory sign, the sensibility of the left side was suddenly impaired, and the woman fell. Her face was shrivelled, pale: she complained of violent pain of the right side of the head. The hemiplegia of the whole left side was imperfect : motions of the heart tumultuous and extensive ; respiration gradually weaker ; anxiety extreme ; death shortly took place. *Dissection.*—The sinuses and superficial vessels of the brain were distended with blood : The substance of it, when cut in layers, displayed bloody points; both it and the cerebellum were very compact. The lateral ventricles contained a little serum. The vertebral canal was not opened ; but a great quantity of blood flowed from within it, on dividing the spinal marrow. The lungs were sound and gorged with blood. Both auricles of the heart were of an enormous size : the right thickened and furnished with numerous radiated columns ; the orifice opening into the right ventricle so large that the two cavities seemed to form but one. There were only two valves ; one situated on the right side, applied against the corresponding wall of the ventricle, and so entangled with the *columnæ carneæ* as to be immoveable. The other valve was almost similarly disposed, but remained free for about four lines at its summit. The left auricle was still more dilated ; but its parietes not thickened. The pulmonary veins and *venæ cavæ* were much enlarged : the left auriculo-ventricular orifice so contracted as scarcely to admit the end of the little finger. The margin of the mitral valves was cartilaginous ; the aorta of a small size. The liver was enlarged ; the stomach so much dilated that the pyloric extremity almost reached the concavity of the ileum. The cavities of the pleura and peritoneum contained a little serum. *Bulletin de la Faculté de Médecine, No.* 8, 1815.—Apoplexy, paralysis, mania, and other diseases indicating lesion of the brain, not unfrequently arise when the heart is suffering from organic affection ; and destroy life. We have most commonly witnessed this termination where the nature of the cardiac lesion was such as to obstruct the return of blood from the head by the jugular veins. EDIT.

*French Method of making Gum-elastic Catheters.*—A firm tissue of silk is woven upon a brass stilette, of the size of the cavity of the instrument to be made. In weaving this tissue, the orifice or eye is left open, and the whole consists of one entire thread. The successive layers of varnish are deposited on the surface of the silken tissue ; and the number of them depends on the size of the instrument. Each coating of varnish undergoes a long process of scouring before the next is put on. Women are employed for this purpose, by Mr. Feburier, principal manufacturer of these instruments, in Paris. They are made of twelve different sizes; and each size is designated by its number; which enables the surgeon to ascertain the progress of a case of stricture. Numbers 1 and 2 are smaller than can be procured in England. *Cross' Sketches of the Medical Schools of Paris.*

We have been informed by a letter from Dr. John Walker, that the Medical Annuitant and Benevolent Society, has constructed the following table of payments for those above 50 years of age, who shall be inclined to become members thereof. In the printed paper which accompanied the Doctor's letter, we find no mention made of a period of ten years being requisite to entitle such contributor to his annuity; and yet it is evident, that without this restriction, the calculation would by no means be proportionate to the respective ages.

*TABLE shewing the Single and Annual Payments for Ten Years, to secure an Annuity of Fifty Pounds during the remainder of a Life* after *Ten Years. The Single and First of the Annual Payments to be made at the beginning of the First Year.*

| Age. | Single Payments, £. | s. | Annual Payments, £. | s. |
|---|---|---|---|---|
| 51 | 209 | 7 | 26 | 12 |
| 52 | 200 | 17 | 25 | 14 |
| 53 | 192 | 4 | 24 | 14 |
| 54 | 183 | 11 | 23 | 14 |
| 55 | 174 | 14 | 22 | 14 |
| 56 | 165 | 18 | 21 | 14 |
| 57 | 156 | 19 | 20 | 12 |
| 58 | 148 | 0 | 19 | 12 |
| 59 | 138 | 19 | 18 | 10 |
| 60 | 129 | 18 | 17 | 10 |

As any discussion of the proposed scheme must be beneficial, we shall subjoin the following observations of Dr. Walker upon the subject.

" The man who joins a Benefit Society with increasing funds from the subscriptions, makes a bad calculation. At the expence of himself and fellow members, he is preparing benefits only for those of future times. In a Society ever verging on insolvency by continual distribution of its funds, the members of the present day are only rendering themselves justice. But the desire of giving permanency to new institutions, by the increase of their funds, is so universally prevalent a disposition with their founders, that this feature, instead of the produced and diffused benefits, is generally considered as constituting the prosperity of the establishments. I was one of the early members putting down their names as desirous of uniting in the Society, and wishing immediately to advance the single payment as an Annuitant. I proposed that the annuity should never exceed fifty pounds; that whatever more the fund might produce, should be given to the benevolent part of the system; but was informed, that others had subscribed on the consideration, that the annuity might be double that sum or more. In what I proposed, I saw in the fixed annuity a certain provision for members not in affluence, to make up for such deficiency in their own means, as has been produced by their devotedness to the relief of their poorer brethren; but this plan not being approved of, I naturally inclined to fall in with the other subscribers for such enlarged annuity as the fund might produce. Though from the fund-making disposition of the early members of societies, I should never expect to obtain the value of my subscription; I was yet disposed to fall in with the plan. Looking, however, forward to the time of annuities becoming due, I anticipate the jealousies and bickerings which are then to rise, from the seeds of discord now preparing. There is no benevolence, no charity, in the annuitant part of the scheme. The principle and spirit of it is purely mercantile. Then why leave to the discretion and disposition of directors the meting out what has been paid for ten years before? If the directors be already entitled to the annuity, they may be tempted to give too much; if not yet entitled, it is their interest to give too little. How will they be able to steer clear of blame? Their business will become most complex.

With my present sentiments, I propose to contribute three guineas per annum to the Benevolent Society; and if the worthy President will bring forward a new temporary or provisional Society, congenially with his first benevolent wishes, to meet cases of distress during the decade of years preceding the beneficient disbursements of the present Society, I shall cheerfully subscribe an equal sum to the evanescent association."

An excellent general account of the splendid Hunterian Museum at Glasgow, has been lately published by Capt. Laskey, including historical and scientific notices of the various objects of art, literature, natural history, anatomical preparations, antiquities, &c. in that celebrated collection.

Mr. Fox, Surgeon Dentist, Argyle Street, will commence his Lectures on the Structure and Diseases of the Teeth, on Friday, the 1st of March, at half past five in the evening, in the Medical Theatre, Guy's Hospital.

Deaths.—Suddenly, Mr. David Price, Apothecary to the London Hospital.—Aged 62, Dr. Thomas Dale, of Devonshire Square, Bishopsgate Street.

